# Supplementary material for: Comparative Effects of Gymnema sylvestre and Berberine on Adipokines, Body Composition, and Metabolic Parameters in Obese Patients: A Randomized Study
Source: Nutrients. 2024 Jul 16;16(14):2284. doi: 10.3390/nu16142284 (PMC11280467; doi:10.3390/nu16142284)
Supplement: Supplementary file 1 [file nutrients-16-02284-s001.zip › nutrients-3084792-supplementary.pdf]

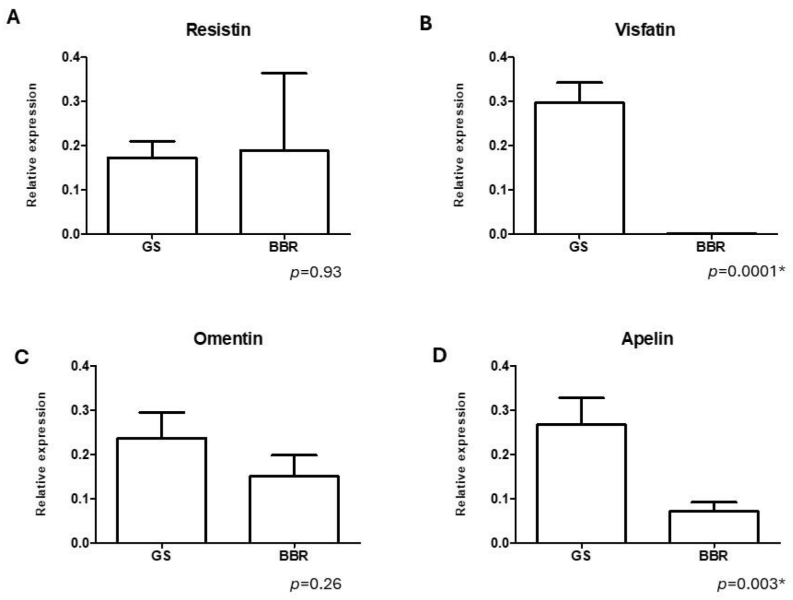

Figure S1. Comparative study of the effect of GS vs BBR on the final values of the gene expression of Resistin, Visfatin, Omentin and Apelin

Table S1. Comparison of baseline vs final anthropometric and physiological measurements in patients treated with *Gymnema sylvestre*

	Basal<br>Mean ± SE	Final<br>Mean ± SE	*p value*
Body Weight (Kg)	93.91±3.88	93.18±3.86	0.10
BMI (Kg/m^2)	36.45±1.23	36.18±1.24	0.11
WHR (cm)	0.95±0.01	0.98±0.01	0.06
Body fat (BF%)	48.06±0.82	46.93±0.91	0.008*
Visceral fat (VF%)	20.92±0.72	20.56±0.78	0.11
SBP (mmHg)	118.40±2.19	119.20±2.44	0.77
DBP (mmHg)	82.80±1.22	83.20±2.30	0.88

SE= Standard error, BMI= Body mass index, WHR= waist-hip ratio, SBP= systolic blood pressure, DBP= Diastolic blood pressure

Table S2. Comparison of baseline vs final biochemical parameters in patients treated with *Gymnema sylvestre*

	Basal<br>Mean ± SE	Final<br>Mean ± SE	*p value*
Fasting glucose (mg/dl)	90.76±1.81	86.42±1.57	0.04*
Insulin (µU/ml)	15.23±2.08	19.43±2.76	0.04*
Glycosylated hemoglobin (%)	5.74±0.19	5.50±0.17	0.0001*
Cholesterol (mg/dl)	207.85±18.42	190.97±39.097.81	0.33
Triglycerides (mg/dl)	131.52±20	127.98±11.43	0.84
LDL (mg/dl)	94.08±4.31	101.64±5.03	0.10
HDL (mg/dl)	41.82±1.75	40.95±2.22	0.5
VLDL (mg/dl)	48.82±4.58	48.36±4.13	0.93

SE= Standard error, LDL= Low-density lipoprotein, HDL= High-density lipoprotein, VLDL=Very low-density lipoprotein

**Table S3. Comparison of baseline vs final anthropometric and physiological measurements in patients treated with** Berberine			
	Basal<br>Mean ± SE	Final<br>Mean ± SE	*p value*
Body Weight (Kg)	88.98±2.26	86±2.21	0.001*
BMI (Kg/m^2)	34.12±0.57	32.98±0.56	0.001*
WHR (cm)	0.92±0.04	0.90±0.01	0.62
Body fat (BF%)	45.36±1	44.07±0.96	0.001*
Visceral fat (VF%)	19.36±0.54	18.32±0.53	0.001*
SBP (mmHg)	116±2.16	112.72±1.53	0.14
DBP (mmHg)	77.20±1	72.80±1.5	0.009*

SE= Standard error, BMI= Body mass index, WHR= waist-hip ratio, SBP= systolic blood pressure, DBP= Diastolic blood pressure

**Table S4. Comparison of baseline vs final biochemical parameters in patients treated with** Berberine			
	Basal<br>Mean ± SE	Final<br>Mean ± SE	*p value*
Fasting glucose (mg/dl)	113.11±2.27	116.44±1.47	0.10
Insulin (μU/ml)	13.22±1.33	15.76±2.70	0.24
Glycosylated hemoglobin (%)	5.58±0.06	5.68±0.05	0.07
Cholesterol (mg/dl)	183.27±5.27	185.46±9.44	0.77
Triglycerides (mg/dl)	170±17.77	141.20±9.06	0.07
LDL (mg/dl)	97.66±4.22	98.96±6.87	0.81
HDL (mg/dl)	34.32±1.99	35.10±1.46	0.68
VLDL (mg/dl)	51.28±4.65	51.39±5.33	0.98

SE= Standard error, LDL= Low-density lipoprotein, HDL= High-density lipoprotein, VLDL=Very low-density lipoprotein

Table S5. Frequency of Gastrointestinal Adverse Effects of Treatments with *Gymnema Silvestre* (GS) and Berberine (BBR)

	Evaluation			
	One month		3 months	
	GS	BBR	GS	BBR
Diarrhea	5	0	2	1
Nausea	3	2	2	3
Constipation	1	0	0	1
Reflux	5	0	2	0
Borborygmus	1	1	0	0
Hemorrhoidal bleeding	0	2	0	2
Pyrosis	0	0	1	1
Polydipsia	6	0	3	2
Colic	1	0	0	0
Xerostomia	3	0	2	0
Dysgeusia	4	0	6	0
Fecal urgency	0	1	0	0
Intestinal spasm	1	0	0	0
Colitis	0	0	0	1
Increased appetite	4	0	1	0
Decreased appetite	1	0	0	0
Gastric fullness	1	0	0	0
Meteorism	0	0	1	0
Gastric empty feeling	0	0	1	0

Table S6. Frequency of somatic, vascular, and sexual adverse effects of treatments with *Gymnema Silvestre* **(GS) vs** Berberine **(BBR)**

	Evaluation			
	One month		3 months	
	GS	BBR	GS	BBR
Somatics				
Headache	5	5	3	3
General discomfort	0	0	1	0
Drowsiness	1	0	0	0
Adynamic	0	1	0	0
Insomnia	2	0	2	0
Vascular				
Vasculopathy	0	0	1	0
Palpitations	1	0	0	0
Venous insufficiency	0	0	1	1
Sexual				
Increased libido	0	0	1	0
Breast turgor	1	0	0	0
